# Antibodies against angiotensin II receptor type 1 and endothelin A receptor are increased in COVID-19 patients

**DOI:** 10.3389/fimmu.2023.1204433

**Published:** 2023-08-08

**Authors:** Jelle R. Miedema, Matthijs L. Janssen, Jan von der Thüsen, Henrik Endeman, Anton W. Langerak, Merel E. Hellemons, Els van Nood, Bas W. A. Peeters, Sara J. Baart, Marco W. J. Schreurs

**Affiliations:** ^1^Department of Pulmonary Medicine, Erasmus Medical Center, Rotterdam, Netherlands; ^2^Department of Intensive Care, Erasmus Medical Center, Rotterdam, Netherlands; ^3^Department of Pathology, Erasmus Medical Center, Rotterdam, Netherlands; ^4^Department of Immunology, Erasmus Medical Center, Rotterdam, Netherlands; ^5^Department of Internal Medicine, Erasmus Medical Center, Rotterdam, Netherlands; ^6^Department of Biostatistics, Erasmus Medical Center, Rotterdam, Netherlands

**Keywords:** COVID-19, endothelin receptor type A antibody, angiotensin II receptor type 1 antibody, antinuclear antibody (ANA), autoimmunity

## Abstract

**Background:**

Increased titers of autoantibodies targeting the G-protein-coupled receptors angiotensin II type 1 receptor (AT1R) and endotelin-1 type A receptor (ETAR) are associated with severe coronavirus disease 2019 (COVID-19) infection. The aim of this study was to determine whether 1) these antibodies are specifically related to COVID-19 disease pathogenesis or increased during any severe respiratory illness, 2) if they are formed during illness, and 3) if they correlate with inflammatory markers or long-term symptoms.

**Methods:**

Antibodies against AT1R, ETAR, and antinuclear antibodies (ANAs) were measured in n=40 prospectively enrolled COVID-19 patients and n=207 COVID-19 patients included in a biobank. Clinical and laboratory findings were prospectively and retrospectively assessed in both cohorts, and results were combined for analysis. The presence of auto-antibodies against AT1R or ETAR in peripheral blood was compared between hospitalized patients with COVID-19 and controls (n=39). Additionally, AT1R and ETAR titers were compared between patients with an unfavorable disease course, defined as intensive care admission and/or death during hospital admission (n=121), to those with a favorable disease course (n=126). A subset of intubated patients with severe COVID-19 were compared to intubated patients with acute respiratory distress syndrome (ARDS) due to any other cause.

**Results:**

Significantly increased AT1R and ETAR antibody titers were found in COVID-19 patients compared to controls, while titers were equal between favorable and unfavorable COVID-19 disease course groups. On ICU, intubated patients with COVID-19 had significantly increased AT1R and ETAR titers compared to patients with ARDS due to any other cause. The titers did not correlate with baseline inflammatory markers during admission or with diffusion capacity, cognitive impairment, or fatigue measured at 3 months follow-up.

**Conclusions:**

In patients hospitalized for COVID-19, antibodies against AT1R and ETAR are increased compared to controls and patients with ARDS due to other causes than COVID-19. The baseline antibody titers do not correlate with inflammatory markers or long-term symptoms in this study.

## 1 Introduction

Several autoantibodies have been related to the disease course of coronavirus disease 2019 (COVID-19), including antibodies against the G-protein-coupled receptors (GPCR) (1). Our prior work published in this journal (2) demonstrated significantly increased autoantibodies against the GPCR angiotensin II receptor type 1 (AT1R) and endothelin receptor type A (ETAR) in patients with COVID-19 with unfavorable disease course compared to mild disease. Together with other recent reports in which COVID-19 patients with severe disease were found to have higher AT1R autoantibody levels than mild disease or controls, this finding suggested that these autoantibodies are associated with disease course (1, 3). However, it is currently unknown whether the increased levels of AT1R and ETAR autoantibodies are specifically seen during COVID-19 infection or if they increase during severe respiratory disease in general due to a dysregulated immune system. Additionally, it remains unknown whether the autoantibody titers increase with time during hospitalization for COVID-19, if they correlate with inflammatory markers, and if they associate with long-term symptoms after hospital discharge.

In the current study, we aimed to address these research questions. We investigated AT1R and ETAR antibody levels in COVID-19 and compared titers with controls and non-COVID-19 acute respiratory distress syndrome (ARDS) patients. Additionally, a subset of intubated patients with severe COVID-19 were compared to intubated patients with ARDS due to any other cause.

Second, we investigated whether AT1R and ETAR antibody titers increase during hospital admission and correlate with known inflammatory markers. Lastly, we assessed the correlation between AT1R and ETAR antibody titers at hospitalization and long-term sequelae of COVID-19 such as symptoms of cognitive impairment, fatigue, and pulmonary function at 3 months after discharge.

## 2 Materials and methods

To assess the titer of AT1R, ETAR, and antinuclear (ANA) antibodies in peripheral blood and to correlate the titers with disease course, we combined data from n=40 prospectively enrolled COVID-19 patients admitted between January and April 2021 with data retrospectively collected from n=207 COVID-19 patients admitted between March and December 2020 to the Erasmus Medical Center in the database EraCORE (COVID-19 Observational Research). Of those, n=207 patients had their baseline sample obtained < 72 hours after admission and were included in analysis and n=66 patients had their baseline serum sample obtained > 72 hours after admission. Both the prospective and retrospective cohort included patients hospitalized for COVID-19 with polymerase chain reaction-confirmed severe acute respiratory syndrome coronavirus 2 (SARS-CoV-2) infection. Patients aged <18 were excluded. To investigate the correlation between antibody titer and COVID-19 disease course, we enriched the retrospective EraCORE cohort with randomly selected COVID-19 patients admitted to the intensive care unit (ICU; n=101, 48.8%). The investigators were blinded for patient selection, as this was done prior to the determination of the titers by a database manager not involved in this study. Clinical and laboratory findings were prospectively and retrospectively assessed in both study cohorts and combined for analysis (n=247 COVID-19 patients). The COVID-19 disease course was defined as unfavorable if patients were admitted to ICU and/or died during hospital admission or as favorable if patients were only treated on the general ward and survived admission.

First, titers of all COVID-19 patients were compared to n= 39 controls from the orthopedic surgery outpatient clinic who provided blood prior to replacement surgery. Exclusion criteria for the control group were systemic inflammation, underlying immunological disease, and concurrent infections. All controls provided their informed consent (METC2011-0409). One control patient was known with cardiovascular disease, one with pulmonary disease, and one with hypertension. Second, AT1R and ETAR titers were compared between COVID-19 patients with a favorable versus unfavorable disease course. Third, antibody titers were compared between a subgroup of COVID-19 patients who were transferred to our hospital at the day of intubation (n=25), which included n=9 patients with a baseline serum sample obtained > 72 hour after admission to titers from ARDS patients with invasive mechanical ventilation (IMV) on ICU that was not related to COVID-19 infection (n=17; IMV controls; **Supplementary Figure S1**). The IMV control patients participated in an ongoing ICU biobank (MEC-2017-0417). All IMV controls had moderate to severe ARDS with a PaO_2_/FiO_2_ (P/F) ratio of <200 due to a non-COVID-19 cause, such as bacterial or viral pneumonia or ARDS secondary to an extra-pulmonary cause. The serological samples of both groups were drawn on the first day of ICU admission, which corresponds with the first day of intubation. Last, the correlation between antibody titers at admission and long-term sequelae of COVID-19 were evaluated in patients from our cohort who also participated in another prospective observational study (n=83). In those patients, additional follow-up information at 3 months from hospital discharge was available, including diffusion capacity for monoxide (DLCO), Montreal Cognitive Assessment (MoCA), and Fatigue assessment scale (FAS) score (4). Ethical approval for the follow-up study was granted under (MEC-2020-0487).

The prospective study was approved by the Institutional Review Board of Erasmus MC (MEC-2020-0902). All participants gave written informed consent. Data from the retrospective patient cohort were obtained from the EraCORE database and biobank, which used an opt-out strategy for admitted COVID-19 patients (MEC-2021-0411). Patients were informed on the use of data and the option to be excluded from the EraCORE database and biobank. Patients who objected to the use of data were excluded from the study.

Laboratory measurements for the presence of ANA, AT1R, and ETAR antibodies were performed at baseline (within 72 h after hospital admission) and on day 7 of admission, if still admitted. Antibody titers on day 7 were unavailable if patients were discharged within a week. All samples were processed and measured at the Laboratory Medical Immunology of the Department of Immunology of the Erasmus MC (Rotterdam, the Netherlands). Anti-AT1R and anti-ETAR were determined using a CE-marked enzyme immuno-assay (EIA), developed at CellTrend GmbH (https://www.celltrend.de/en/elisa/in-vitro-diagnostika-human/), according to the manufacturer’s instructions. For antibodies against AT1R and ETAR, a concentration of 10 U/mL was considered the cutoff value (10–17 U/mL borderline and >17 U/mL positive) in line with the manufacturer’s recommendation and our previous work (2). Anti-nuclear antibodies (ANAs) were determined using classical indirect immunofluorescence on HEp2 cells (Inova, San Diego, CA), at 1:80 serum dilution, according to standard protocol. Only nuclear immunofluorescence patterns were considered ANA positive.

### 2.1 Statistical analysis

Differences between the three patients groups in AT1R and ETAR antibody titer were calculated using independent samples Kruskal–Wallis tests. Comparisons in continuous variables between two specific groups were analyzed with Mann–Whitney U tests. For categorical variables, chi-square test (or Fisher’s exact test if <5 cases per cell) were used. Continuous data are presented as median with interquartile range (IQR). Categorical variables are reported as number with percentages. Correlations between baseline antibody titers and long-term data 3 months after discharge were compared with Spearman’s Rho. *p*-values < 0.05 were considered statistically significant. Statistical analysis was performed with R version 4.2.1 and IBM SPSS Statistic version 28.0.1.0.

## 3 Results

Baseline characteristics of controls, IMV controls, and total COVID-19 patients are shown in **Table 1**. In summary, COVID-19 patients and IMV controls were more often men and known with cardiovascular disease, hypertension, and renal disease compared to controls (**Table 1**). The main cause for respiratory failure in the IMV control group was bacterial pneumonia (n=9), viral pneumonia (n=2), aspiration pneumonia (n=3), and secondary to extra-pulmonary cause (n=3).

**Table 1:** Characteristics of different cohorts.

	controls (n=39)	IMV controls (n=17)	COVID-19 patients (n=247)	P-value^1^	P-value^2^
Males	12 (31)	14 (82)	163 (66)	<0.001	0.009
Age (years)	60 (58 – 62)	62 (56 - 74)	63 (55 – 72)	0.187	0.177
BMI (kg/m2)	–	26.1 (22.8 – 28.8)	27.8 (24.8 – 31.7)		0.167
Obesity	–	2 (12)	75 (35)		0.342
Smoking status					0.006
Never	–	4 (24)	77 (31)		
Former	–	7 (41)	51 (21)		
Current	–	2 (12)	11 (5)		
Unknown	39 (100)	4 (24)	108 (44)		
Diabetes Mellitus				0.221	0.120
Uncomplicated	4 (10.3)	3 (18)	54 (22)		
End-organ damage	0	1 (6)	13 (5)		
Cardiovascular disease	1 (3)	5 (30)	57 (23)	0.009	0.040
Hypertension	1 (3)	6 (35)	133 (55)	<0.001	<0.001
Pulmonary disease	1 (3)	7 (41)	51 (21)	0.002	0.278
Renal disease	0	2 (12)	39 (16)	0.027	0.016
Transplant recipient	0	2 (12)	20 (8)	0.148	0.390
AT1R baseline (U/mL)	8 (6 – 12)	6 (4 - 12)	10 (8 – 15)	0.002†	0.001
AT1R baseline positive	3 (8)	3 (18)	39 (16)	0.381	0.314
ETAR baseline (U/mL)	7 (5 - 10)	7 (4 - 10)	12 (9 – 16)	<0.001*,†	<0.001
ETAR baseline positive	3 (8)	2 (12)	48 (20)	0.150	0.056
ANA positive baseline^±^	3 (8)	3 (18)	70 (30)	0.272	0.109

Continuous data are presented as median with interquartile range (IQR). Categorical variables are reported as number with percentages. Categorical variables are compared between groups with Chi-square test or Fishers’ exact test if <5 cases per cell. Continuous data are compared with the Mann-Whitney U test for comparison between 2 groups and Kruskall-Wallis test for comparison between 3 groups. After statistically significant Kruskall-Wallis tests, post-hoc Dunn test with Holms adjustment for multiple testing were performed. Siginificant post-hoc tests are indicated with * or ^†^: *Post hoc P-value for controls vs. COVID-19 patients <0.05, ^†^post-hoc P-value for IMV controls vs. COVID-19 patients <0.05. 1: Compared between the 3 subgroups. 2: Compared between patients with and without COVID-19, i.e. grouped the two control groups together.

**Figure 1:**
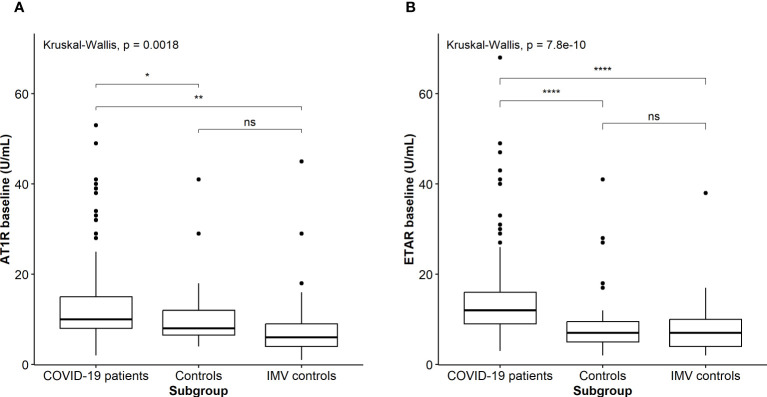
**(A)** Comparison of baseline AT1R titers between patients with COVID-19 and the control groups. Asterisks indicate significant *p*-values. *p < 0.05 and **p < 0.01. NS, not significant. **(B)** Comparison of baseline ETAR titers between patients with COVID-19 and the control groups. Asterisks indicate highly significant *p*-values: ****p < 0.0001. NS, not significant.

No significant differences were observed in positive ANA titer between total COVID-19 patients, controls, and IMV controls (p=0.272), or between COVID-19 patients with favorable (n=37; 29%) and unfavorable disease course (n=33; 27%) (*p*= 0.842). First, we compared AT1R and ETAR titers between patients with COVID-19 and the control groups. We found a significantly increased baseline AT1R titer in COVID-19 patients (11; IQR, 8–16) compared to controls (10; IQR, 8–15) and IMV controls (6; IQR, 4–12) (*p*=0.002), but not between controls and IMV patients (**Figure 1A**). In addition, we found an increased baseline titer of ETAR in COVID-19 patients (12; IQR, 9–16), compared to controls (7; IQR, 5–10) and IMV controls (7; IQR, 4–10) (*p*<0.001). Again, no difference was found in ETAR titers between controls and IMV controls (**Figure 1B**).

**Figure 2:**
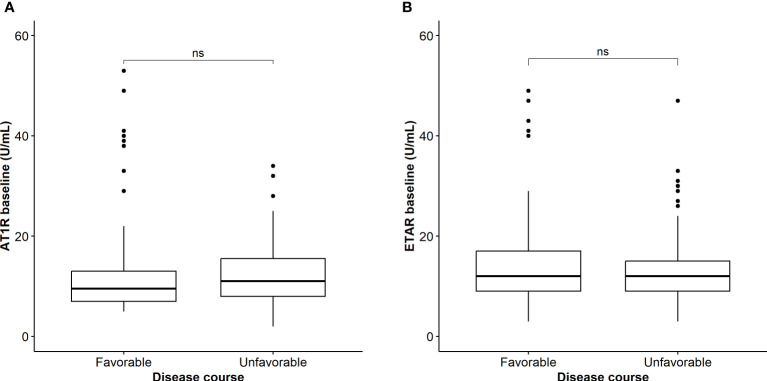
**(A)** Comparison of baseline AT1R titers between patients with COVID-19 with favorable and unfavorable outcome. NS, not significant. **(B)** Comparison of baseline ETAR titers between patients with COVID-19 with favorable and unfavorable outcome. NS, not significant.

Subsequently, to evaluate the association between antibody titers and disease course in COVID-19, we compared titers between COVID-19 patients with favorable and unfavorable disease course as earlier defined. Baseline characteristics are shown in **Table 2**. In summary, patients with an unfavorable disease course were significantly more often men, but there was no difference in age, body mass index, duration of symptoms, or cardiovascular comorbidity compared to patients with favorable disease course. The unfavorable group had higher baseline inflammatory markers (leukocytes, CRP, ferritin, and D-dimer), had received treatment with dexamethasone less often, but treatment with methylprednisolone in high dose more often, and had a higher incidence of pulmonary embolism (**Table 2**). We found no significantly different AT1R and ETAR antibody titers between the favorable and unfavorable COVID-19 disease outcome groups in this cohort (**Figures 2A, B**). To correct for relevant confounders and baseline differences, multivariable analysis, including steroid use, age, sex, BMI, and several inflammatory markers into account, AT1R and ETAR antibody titers, and an interaction term for steroid use and antibody titer, was performed. Baseline AT1R and ETAR antibody titers did not associate with COVID-19 disease outcome (**Supplementary Table S1**). Having a positive ETAR titer at baseline did not associate with favorable outcome in multivariable analysis (*p*=0.09). Titers of AT1R and ETAR antibodies of the seventh day of hospitalization were available in n=13 patients with favorable disease course and n=80 patients with unfavorable disease course. We found no significant antibody increase between day 1 and day 7, nor any difference in AT1R and ETAR antibody titer on day 7 of admission between favorable and unfavorable outcome groups (**Table 2**). Having a combined positive AT1R and ETAR antibody titer (n=30) did not correlate with ICU admission or mortality compared to COVID-19 patients who did not have both antibodies (n=213; *p*=0.57 and *p*=0.78, respectively).

**Table 2:** Characteristics of patients with COVID-19 with favorable and unfavorable outcome.

	Favorable disease course (n=126)	Unfavorable disease course (n=121)	P-value
Males	73 (58)	90 (74)	0.006
Age (years)	63 (55 – 72)	63 (55 – 72)	0.887
BMI (kg/m^2^)	27.1 (24.3 – 30.9)	28.4 (25.1 – 32.4)	0.079
Obesity	32 (25)	43 (36)	0.186
Diabetes Mellitus			0.712
Uncomplicated	25 (20)	29 (24)	
End-organ damage	7 (6)	6 (5)	
Cardiovascular disease	35 (28)	22 (18)	0.074
Hypertension	69 (55)	64 (53)	0.949
Pulmonary disease	24 (19)	27 (22)	0.526
Renal disease	23 (18)	16 (13)	0.278
Transplant recipient	14 (11)	6 (5)	0.076
Duration of symptoms at admission (days)	7 (4 – 10)	8 (6 - 12)	0.186
Leukocytes (10^9^ /L)	5.8 (4.5 – 7.6)	8.1 (6.0 - 10.9)	<0.001
CRP (mg/L)	65.5 (37.3 – 111.0)	115.5 (62.4 – 196.0)	<0.001
Ferritin (µg/L)	566 (244 - 981)	830 (447 - 1566)	0.002
IL-6 (pg/mL)	29.5 (16.3 – 125.3)	88.0 (27.5 – 266.5)	0.094
D-dimer (mg/L)	0.87 (0.42 – 1.63)	1.34 (0.78 – 3.90)	0.006
AT1R baseline (U/mL)	10 (7 - 14)	12 (8 - 16)	0.124
AT1R baseline positive	17 (14)	22 (18)	0.260
ETAR baseline (U/mL)	12 (9 - 18)	12 (9 – 15.5)	0.229
ETAR baseline positive	32 (25)	16 (13)	0.022
ANA positive baseline^±^	37 (29)	33 (27)	0.842
AT1R day 7 (U/mL)*	10 (8.5 - 13)	12 (9.3 - 16)	0.207
AT1R day 7 positive*	2 (15)	17 (21)	0.627
ETAR day 7 (U/mL)*	13 (11 – 23.5)	13 (10 - 16)	0.544
ETAR day 7 positive*	4 (31)	14 (18)	0.270
ACE- or AT2 inhibitor use	19 (15)	15 (12)	0.541
Dexamethasone use (6 mg/day)	93 (74)	71 (58)	0.015
Methylprednisolone use (1000 mg/ day)	7 (6)	20 (17)	0.003
IL-6 inhibitor use (tocilizumab 600 mg once)	5 (4)	8 (7)	0.352
Pulmonary embolism	2 (2)	32 (26)	<0.001
ICU admission	0	106 (88)	–
Invasive mechanical ventilation	0	93 (77)	–
Deceased	0	32 (26)	–

Continuous data are presented as median with interquartile range (IQR). Categorical variables are reported as number with percentages. Continuous data are compared with the Mann-Whitney U test for comparison between 2 groups and Kruskall-wallis test for comparison between 3 groups. Categorical variables Chi-square test or Fishers’ exact test if <5 cases per cell. Comorbidities are difined as in the Charlson Comorbidity Index. Pulmonary Disease: Asthma, Chronic Obstructive Pulmonary Disease or Obstructive Sleep Apnea in medical history. Cardiovascular disease: Myocardial infarction, Congestive heart failure, Cerebrovascular Accident or Peripheral Vascular Disease in medical history. *Day 7 samples are obtained in n=13 patients with favorable disease course and n=80 patients with unfavorable disease course. ±ANA positivity is defined as weakly positive, positive or strongly positive. BMI, Body Mass Index; CRP, C-Reactive Protein; IL, Interleukin; ICU, Intensive Care Unit; AT1R, Angiotensin II receptor type 1; ETAR, Endothelin A receptor; ACE, Angiotensin Converting Enzyme; AT2, Angiotensin II; ANA, Anti-Nuclear Antibody.

**Figure 3:**
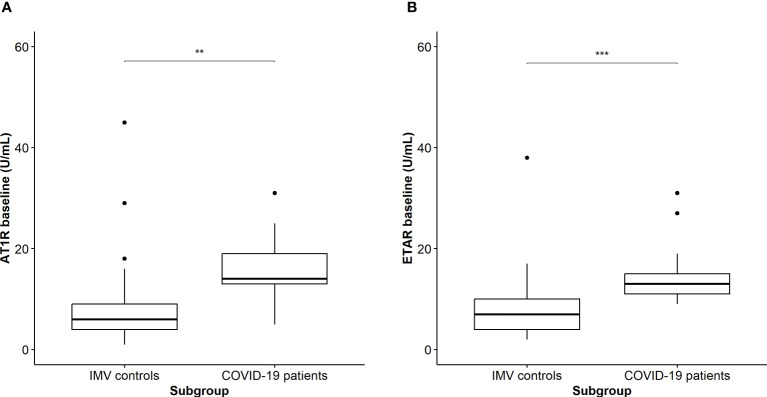
**(A)** Comparison of AT1R titers on the day of intubation between patients with COVID-19 and IMV controls. Asterisks indicate significant p-values: **p < 0.01 **(B)** Comparison of ETAR titers on the day of intubation between patients with COVID-19 and IMV controls. Asterisks indicate significant p-values: ***p < 0.001.

Next, we compared AT1R and ETAR titers between a subgroup of COVID-19 patients with an available baseline blood sample taken on the day of intubation (n=25) and compared this with blood samples drawn at ICU admission in ARDS patients without COVID-19 (IMV controls; n=17). Baseline characteristics of both groups are shown in **Table 3**. The groups were comparable concerning age, sex, and body mass index. Intubated COVID-19 patients more often had hypertension (67% versus 35%; *p*=0.047), but the number of patients with diabetes, cardiovascular, and pulmonary comorbidity were comparable between the groups. In contrast to the IMV controls, COVID-19 patients were treated with dexamethasone (40%) or methylprednisolone (6.3%) (**Table 3**). We found significantly increased AT1R (14.0; IQR, 12.5–19.5) in COVID-19 patients compared to IMV controls (6.0; IQR, 4.0–12.5) (*p*=0.002) and significantly increased ETAR (13.0; IQR, 10.5–15.0) compared to IMV controls (7.0; IQR, 4.0–10.0) (**Figures 3A, B**, *p*<0.001). There was no difference in ANA positivity between the groups (**Table 3**, *p*=0.490).

**Table 3:** Characteristics of Invasive Mechanical Ventilation (IMV) control patients and intubated COVID-19 patients.

	IMV controls (n=17)	Intubated COVID-19 patients (n=25)	P-value
Males	14 (82.4)	22 (88.0)	0.608
Age (years)	62.0 (56.0-74.0)	63.0 (53.5-71.5)	0.691
BMI (kg/m2)	27.1 (24.3 – 30.9)	28.4 (25.1 – 32.4)	0.079
Obesity	26.1 (22.8 – 28.8)	26.3 (25.2 – 32.3)	0.445
Diabetes Mellitus			0.280
Uncomplicated	3 (17.6)	2 (8.0)	
End-organ damage	1 (5.9)	0 (0)	
Cardiovascular disease	5 (29.4)	4 (16.0)	0.446
Hypertension	6 (35.3)	16 (66.7)	0.047
Pulmonary disease	7 (41.2)	8 (32.0)	0.542
Renal disease	2 (11.8)	0 (0)	–
Transplant recipient	2 (11.8)	1 (4.0)	0.556
Admission days until intubation	3 (1-8.5)	1 (1-4)	0.164
Leukocytes (10^9^ /L)	6.5 (4.4-16.2)	9.8 (7.4-12.5)	0.537
CRP (mg/L)	61.5 (14.8-168.5)	160.7 (95.5-221.6)	0.045
Ferritin (µg/L)	–	1276 (758-1755)	–
IL-6 (pg/mL)	–	120.0 (35.0-174.0)	–
D-dimer (mg/L)	–	2.71 (0.99-10.3)	–
AT1R baseline (U/mL)	6.0 (4.0-12.5)	14.0 (12.5-19.5)	0.002
AT1R baseline positive	3 (17.6)	8 (32.0)	0.299
ETAR baseline (U/mL)	7.0 (4.0-10.0)	13.0 (10.5-15.0)	<0.001
ETAR baseline positive	2 (11.8)	4 (16.0)	1.0
ANA positive baseline^±^	3 (17.6)	7 (28.0)	0.490
Dexamethasone use (6 mg/day)	–	10 (40.0)	–
Methylprednisolone use (1000 mg/ day)	–	1 (6.3)	–
IL-6 inhibitor use (tocilizumab 600 mg once)	–	0 (0)	–

Comparison of patients undergoing Invasive Mechanical Ventilation (IMV) based on COVID-19 status, including only those in the COVID-19 group, that were admitted to the ICU on the first day of admission in our center. This means that the baseline samples represent the data on the day of ICU admission. Continuous data are presented as median with interquartile range (IQR). Categorical variables are reported as number with percentages. Continuous data are compared with the Mann-Whitney U test for comparison between 2 groups. Categorical variables Chi-square test or Fishers’ exact test if <5 cases per cell. Comorbidities are defined as in the Charlson Comorbidity Index. Pulmonary Disease: Asthma, Chronic Obstructive Pulmonary Disease or Obstructive Sleep Apnea in medical history. Cardiovascular disease: Myocardial infarction, Congestive heart failure, Cerebrovascular Accident or Peripheral Vascular Disease in medical history. ±ANA positivity is defined as weakly positive, positive or strongly positive. BMI, Body Mass Index; CRP, C-Reactive Protein; IL, Interleukin; ICU, Intensive Care Unit; AT1R, Angiotensin II receptor type 1; ETAR, Endothelin A receptor; ACE, Angiotensin Converting Enzyme; AT2, Angiotensin II; ANA, Anti-Nuclear Antibody.

It is currently unknown if the formation of AT1R and ETAR antibodies is related to a hyper-inflammatory state, often seen in COVID-19 patients with an unfavorable disease course. We therefore correlated baseline AT1R and ETAR antibodies with baseline CRP, interleukin (IL) 6, ferritin, and D-dimer. While AT1R and ETAR titers correlated with each other (R^2 ^= 0.722, *p*<0.001), we found no significant and relevant correlation between AT1R or ETAR and the inflammatory markers (**Supplementary Figure S2**). Last, because it is unknown if autoimmunity might have a role in long-term post-COVID-19 symptoms, we correlated baseline AT1R and ETAR titers with available data from n=86 COVID-19 patients that were collected during an outpatient follow-up visit 3 months after hospital discharge. We found no significant correlation between AT1R or ETAR titers at admission or day 7 and pulmonary function measured by diffusion capacity (DLCO), cognitive impairment measured by the Montreal Cognitive Assessment (MoCA), or fatigue measured by the Fatigue Assessment Scale (FAS) (**Supplementary Table S2**).

## 4 Discussion

In summary, we found increased AT1R and ETAR titers in COVID-19 patients compared to controls, but in contrast to our previous report (2), we did not find an increased AT1R and ETAR titer in an unfavorable compared to a favorable COVID-19 disease course. The equal distribution of ANA in the groups supports the hypothesis of a specific autoimmune response. Additionally, the titers of AT1R and ETAR did not increase over the course of the first week of hospitalization. On ICU, intubated patients with COVID-19 had significantly increased baseline AT1R and ETAR titers compared to patients with ARDS from any other cause (without COVID-19). In COVID-19 patients, AT1R and ETAR titers did not correlate with baseline inflammatory markers or diffusion capacity, cognitive impairment, or fatigue measured at 3 months follow-up.

The generation of autoantibodies targeting GPCRs, including AT1R and ETAR, has been demonstrated during COVID-19, but their contribution to disease pathogenesis is not well understood (5). Several studies found increased levels of AT1R and ETAR in severe COVID-19 disease course compared to mild disease or controls (1–3). However, because COVID-19 disease outcome was compared to controls, it is unknown whether the increased AT1R and ETAR autoantibodies are specifically related to COVID-19 pathogenesis or the result of severe respiratory illness in general. To the best of our knowledge, this study is the first to compare AT1R and ETAR antibodies in intubated COVID-19 patients to intubated ARDS patients without COVID-19. We found an increase in AT1R and ETAR titers in intubated patients with COVID-19 compared to intubated control patients with other respiratory disease, which suggests that these autoantibodies may be specifically related to COVID-19 pathogenesis.

Both AT1R and ETAR are expressed in several tissues in the human body, including the vascular endothelium, immune cells, and pulmonary tissue (6). Antibodies targeting the AT1R can activate the angiotensin II type 1 receptor, which regulates water–salt balance and blood pressure (7). Antibodies against ETAR have neutrophil chemotactic properties (8). The increased serum levels and downstream pro-inflammatory and vasoconstrictive effects of anti-AT1R and anti-ETAR have been associated with vascular (non-HLA dependent) renal-allograft rejection (7, 9). Furthermore, increased AT1R and ETAR antibodies have been demonstrated in systemic sclerosis and connective tissue disease-associated pulmonary arterial hypertension (10), cardiovascular disease, hypertension, and pre-eclampsia (6), which suggests that these antibodies are related to inflammation and vascular function.

We found increased AT1R and ETAR antibodies in COVID-19 patients compared to controls, but surprisingly and in contrast with previous studies, we did not find increased titers in unfavorable compared to favorable COVID-19 disease (1–3). Although the reason for this discrepancy with previous findings remains unknown, there are differences between patients cohorts to take into account. First, the admitted COVID-19 patients in this study were treated differently as corticosteroids, and interleukin-6 antagonists are now standard of care for hospitalized patients with hypoxemia. In contrast, only 17% of COVID-19 patients were treated with corticosteroids in our previous study (2). This treatment may have influenced the antibody titers in the current study cohort. Second, the current study included more patients in both groups. Third, unfavorable disease course was defined as ICU-admission or death. However, during the pandemic, an increasing proportion of patients were treated with non-invasive respiratory support (NIRS) such as high-flow nasal oxygen outside ICU setting. Those patients were classified as “favorable” disease course if they survived hospitalization without ICU admission, whereas they would have been considered as “unfavorable” in hospitals that did not use NIRS outside ICU. It is possible that patients with NIRS classified as favorable outcome contributed to the discrepancy with previous studies when comparing unfavorable with favorable COVID-19 disease. Unfortunately, our data on the use of NIRS are insufficient for sensitivity analyses. Last, other COVID-19 subtypes were circulating during the current study compared to earlier work, which may result in altered immunological responses in patients.

It is unknown if AT1R and ETAR antibodies are generated *de novo* and increase in concentration during COVID-19 infection. We therefore investigated the course of anti-AT1R and anti-ETAR serum titers and compared samples taken on day 1 of hospital admission with available samples from day 7 in a subgroup of patients who were still admitted, but found no change of the titers. Because the median duration of symptoms in patients at admission was 8 days (IQR, 5–11), the increase in AT1R and ETAR antibody titer in COVID-19 patients may occur very early during the disease course. We were not able to determine antibody titers in patients who were discharged or died during the first week after admission. This may have affected our assessment of the evolution of antibody titers in both directions, so we are unable to draw definitive conclusions in this matter.

It has been hypothesized that increased AT1R and ETAR antibodies in severe COVID-19 disease could be the consequence of endothelial damage in a pro-inflammatory environment (11). We found no correlation between baseline inflammatory markers and AT1R and ETAR antibodies, which suggests that the formation of these antibodies may be more related to vascular endothelial damage itself, independent of the systemic inflammatory cascade. In line with previous results in healthy donors and autoimmune diseases (8), the AT1R and ETAR antibodies correlated with each other in COVID-19 patients.

A study investigating the presence of antibodies to GCPRs in patients with long-term post-COVID-19 symptoms found a high prevalence of two antibodies, including AT1R in 29 (90%) of 31 participants, suggesting that these autoantibodies may be involved in the pathogenesis of long-term COVID-19 symptoms (12). In the current study, however, we did not find a correlation between baseline AT1R end ETAR antibody titers and long-term lung function, cognitive functioning, or fatigue.

Major strengths of our study are the use of multiple control groups and a large group of patients with COVID-19. However, we address some limitations. The majority of included patients were assessed retrospectively because the prospective inclusion of new patients was limited due to the low incidence of new infections during the inclusion period. This, therefore, warrants cautious interpretation of the data. Additionally, baseline blood samples were drawn within the first 72 h after admission. Any treatment with corticosteroids may have affected baseline antibody titers. We found no differences in positive AT1R or ETAR antibodies between patients and controls or favorable and unfavorable disease course using the cutoff value >17 U/mL in multivariable analysis, and overall differences in titers were small. We were not able to measure AT1R and ETAR antibodies at 3 months follow-up to investigate if increased titers persist and whether they correlate with long-term symptoms. Furthermore, we used healthy subjects and patients with respiratory failure due to non-COVID-19 causes as controls. The addition of another large control group specifically consisting of patients with other viral pneumonia with comparable characteristics and disease severity would further have strengthened our understanding of the COVID-19 specificity of AT1R and ETAR auto-antibody levels. While included COVID-19 patients and IMV controls were more often men, the majority of the control group were female participants. This significant difference may have confounded the results. Last, although our findings add to the understanding of deregulated autoantibody production in COVID-19 pathophysiology, the clinical relevance may be limited. In summary, the data presented in this study show increased AT1R and ETAR antibodies in COVID-19 patients compared to controls and in severe COVID-19 compared to patients with non-COVID-19 ARDS. Both autoantibodies are not associated with baseline inflammatory markers and do not correlate with lung function (DLCO), fatigue, or cognitive function measured 3 months after admission.

## Data availability statement

The raw data supporting the conclusions of this article will be made available by the authors upon reasonable request.

## Ethics statement

The studies involving human participants were reviewed and approved by Medical Ethics Review Committee (MERC) Erasmus Medical Center, Rotterdam, the Netherlands. The patients/controls provided their written informed consent to participate in this study. Data from the retrospective patient cohort were obtained from the EraCORE database and biobank which used an opt-out strategy for admitted COVID-19 patients. Participants were informed on the use of data and the option to be excluded from the EraCORE database and biobank. Patients who objected to the use of data were excluded from the study.

## Author contributions

JM, MJ, HE, SB, JT, and MS made substantial contributions to the conception and acquisition, drafting, and analysis and interpretation of data for the work. All listed authors contributed to data acquisition, analysis and interpretations, and made critical revisions. All authors agree with accountability for all aspects of the work and ensure the accuracy or integrity of any part of the work. All authors contributed to the article and approved the submitted version.

## Supplementary material

Supplementary Figure 1 Visual representation of the different cohorts used in this analysis. IMV: invasive mechanical ventilation.

Supplementary Figure 2 **(A)** Correlations between baseline AT1R and other inflammatory markers in COVID-19 patients. **(B)** Correlations between baseline ETAR and other inflammatory markers in COVID-19 patients.
