# Supplementary figures and images for: Antibodies against angiotensin II receptor type 1 and endothelin A receptor are increased in COVID-19 patients
Source: Front Immunol. 2023 Aug 8;14:1204433. doi: 10.3389/fimmu.2023.1204433 (PMC10446834; doi:10.3389/fimmu.2023.1204433)

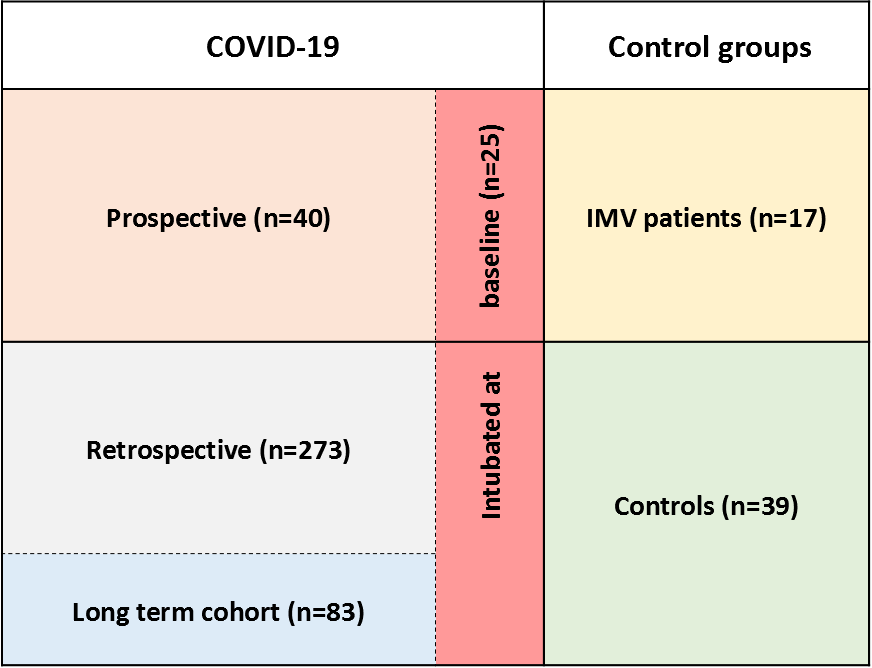

Supplement: Supplementary Figure 1 — Visual representation of the different cohorts used in this analysis. IMV: invasive mechanical ventilation. [file Image_1.tif]

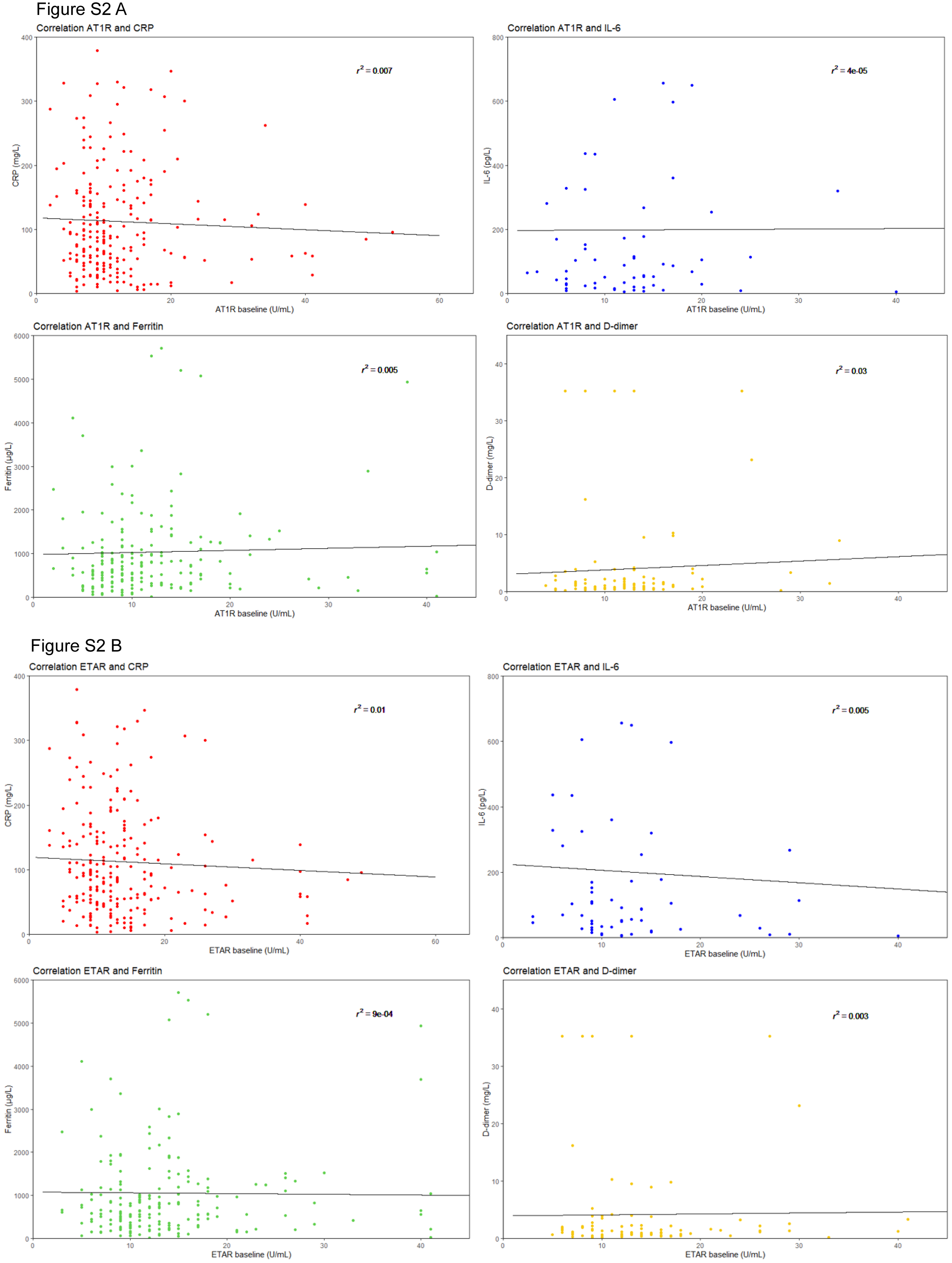

Supplement: Supplementary Figure 2 — (A) Correlations between baseline AT1R and other inflammatory markers in COVID-19 patients. (B) Correlations between baseline ETAR and other inflammatory markers in COVID-19 patients. [file Image_2.tif]
